# Effect of Mo Concentration on the Microstructure Evolution and Properties of High Boron Cast Steel

**DOI:** 10.3390/ma13040975

**Published:** 2020-02-21

**Authors:** Zhiguo Chen, Sen Miao, Lingnan Kong, Xiang Wei, Feihong Zhang, Hongbin Yu

**Affiliations:** 1 School of Materials Science and Engineering, Central South University, Changsha 410083, China; 2 Department of Mechanical and Electrical Engineering, Hunan University of Humanities, Science and Technology, Loudi 417000, China; undyde@163.com (L.K.);; 3 Valin Lianyuan Steel Co Ltd., Loudi 417009, China; hbyu8211@sina.com

**Keywords:** molybdenum, high boron cast steel, borides, mechanical properties, wear resistance

## Abstract

The microstructure evolution, mechanical properties, and tribological properties of high boron cast steel (HBCS) with various Mo concentrations are investigated. The results indicate that Mo addition can significantly modify the microstructure and enhance the comprehensive properties. With the increase of Mo concentration, borides change from the original fish-bone Fe-rich and Cr-rich M_2_B to dendritic Fe-rich M_2_B, blocky and cluster-like Cr-rich M_2_B, and grainy Mo-rich M_2_B. The hardness of HBCS increases gradually with the increase of Mo content due to the solid solution strengthening and the refinement of M_2_B. It can be found that all the samples exhibit quasi-cleavage, but the impact toughness increases firstly and reaches the maximum value when the concentration of Mo is 2.10 wt.%, which is the result of the dispersive distribution of M_2_B rather than the original fish-bone M_2_B. Subsequently, the impact toughness begins to decrease as the concentration of Mo further increases because of the extensive formation of grainy Mo-rich M_2_B at the grain boundary. Meanwhile, the wear results reveal that the average friction coefficient and wear ratio decrease with the increase of Mo content, and the wear mechanism changes from abrasive wear and adhesive wear to abrasive wear when the concentration of Mo exceeds 2.10 wt.%.

## 1. Introduction

Wear, as one of the most common failure modes of metal materials, has received much attention due to its ubiquity in industrial fields and consequential tremendous economic loss [1,2,3]. Moreover, with the rapid development of industrial technology, the wear of mechanical parts becomes increasingly severe. Therefore, materials with superior wear resistance are urgently demanded [4,5].

As is well known, to improve wear resistance, the wear-resistant materials have undergone a process of evolution from white cast iron, high manganese steel, alloyed white cast iron such as nickel-hard cast iron, and high chromium cast iron (HCCI) to high speed steel (HSS) [6,7,8,9,10]. Furthermore, correspondingly, their reinforcing phases have experienced the development from Fe_3_C and M_7_C_3_ to MC and M_2_C (M represents alloying elements such as Cr, Mo, V, Ni, and W) since, generally speaking, the wear resistance of materials is proportional to the hardness of the materials. Unfortunately, to achieve better wear resistance in this way, on the one hand, a large amount of carbon is needed for forming the carbide reinforcing phases, consequently resulting in the formation of the high-carbon martensitic matrix with poor toughness [11,12,13]. On the other hand, it is easy to know that the harder the carbide is, the more expensive metals are needed, which intensely increases the cost of wear-resistant materials [14,15,16].

High boron cast steel (HBCS), which takes borides (Fe_2_B) as reinforcing phases, emerges as required [17,18,19]. The extraordinary advantage of HBCS is that hardness and toughness can be controlled by the content of boron and carbon, respectively [20,21,22]. Resulting from a competitive hardness of borides, HBCS possesses a similar wear resistance to HCCI and HSS, which brings about broad application prospects. Nevertheless, the borides are generally formed in a continuous network manner because iron has a very low solubility for boron, which greatly reduces the continuity of the matrix and damages the toughness of the material [23,24,25]. Moreover, Fe_2_B has intrinsic brittleness due to the weak B-B bond along the [002] direction, leading to a strong susceptibility to cracking, which significantly brings down the toughness of HBCS [26].

Alloying is a direct and effective means to improve the properties of alloys [27]. In order to improve the toughness of HBCS, Cr, W, Cu, Ni, and other elements have been widely investigated [21,28,29,30,31,32,33,34,35]. Ma [21] and Lentz [28] et al. pointed out that with an increase of Cr, the morphology of Fe_2_B changes from a blocky shape to a rod-like shape. Besides, hardness increases, and the fracture toughness increases firstly and then decreases. Wei et al. [29] investigated the effect of alloying elements M (M = Ti, V, Cr, Mn, Co, Ni, and Cu) on the mechanical, electronic, and magnetic properties of Fe_2_B by first-principles calculation. They revealed that all the alloying elements except Mn can enhance the ductility of Fe_2_B. Yi et al. [30,31] suggested that Cu and Ni are able to enhance the hardenability of the matrix, promote the formation of martensite, and improve the hardness and impact toughness of the alloy, but the excessive Cu will damage the hardness and wear resistance of the material due to the formation of pearlite. Based on the Mo alloyed oriented bulk Fe_2_B crystal [32] and Fe-5 wt.% B alloy [33], Huang and Jian revealed that Mo showed the capacity to improve the toughness of Fe_2_B. Most recently, Yi et al. [34] investigated the abrasive wear behavior of Fe-B alloys containing Mo and suggested that the addition of Mo is beneficial to the improvement of the abrasion resistance of Fe-B alloy. However, little work has been involved in the effect of Mo concentration on the microstructure evolution and comprehensive properties of HBCS so far. The underlying mechanism for the microstructure regulation by Mo addition is still unclear.

The influence of Ce on microstructure of high boron steel has been studied by our group [35]. The results indicate that the primary austenite grains can be greatly refined by the addition of Ce. The impact toughness and wear resistance are enhanced with the modification of boron carbide morphology. In this study, the microstructure evolution and properties of Ce-bearing HBCS with different Mo content are systematically investigated for developing high performance high boron wear-resistant cast steel.

## 2. Materials and Methods

**Figure 1:**
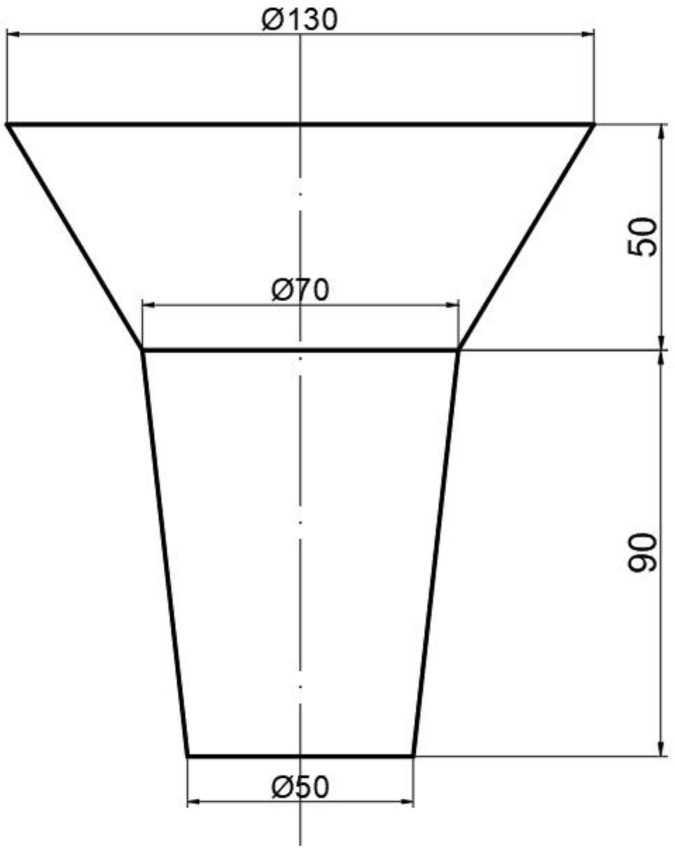
Shape and dimension of the ingot (unit: mm).

The alloys were prepared in a vacuum induction furnace with charge materials of pig iron, ferrochrome, ferromolybdenum, ferrosilicon, ferromanganese, ferroboron, and cerium. The melt was superheated to 1823 K and poured into the mold at 1723-1753 K. All samples were cut from the lower part of the cast ingot, and the shape and dimension of the ingot are shown in Figure 1. The samples were heat treated at 1273 K for 2 h in an electrical resistance furnace, quenched in water, and then tempered at 472 K for 2 h followed by air cooling.

The chemical compositions were analyzed by inductively coupled plasma optical emission spectrometer (ICP-OES), which are presented in Table 1. For convenience, the 4 samples were named according to the Mo concentration as A0, A1, A2, and A3, respectively. X-ray diffraction (XRD), which was performed on a D/max 2550 diffractometer with copper Kα radiation coupling continuous scanning, was carried out for phase analysis. The specimens were scanned in the angular 2θ range from 20°to 85°, and the scanning speed was 2° per minute. An optical microscope (OM, Leica DM4M, Leica, Wetzlar, HE, Germany) and scanning electron microscope (SEM, Sirion-200 and FEI Quanta-200, FEI, Hillsboro, OR, USA) were adopted for microstructure observation. The Sirion-200 was used for secondary electron imaging (SE), and the back scattered electron imaging (BSE) was performed on FEI Quanta-200. The accelerating voltage of both scanning electron microscopes was 20 kV. An electron probe micro-analyzer (EPMA, JXA-8230, JEOL, Akishima-shi, Tokyo, Japan) was employed for elemental analysis. The samples were polished, followed by etching with 4 vol% natal solution for metallographic observation.

The hardness of the samples was measured by a Rockwell hardness tester (HR-150A, Huayin, Yantai, China) and a Vickers hardness tester (HMV-2T, Shimadzu, Tokyo, Japan). The indenter load was 100 g, and the holding time was 10 s in the micro-hardness test. Seven hardness measurements were operated for each sample, and an average value was taken after deleting the maximum and minimum value. The impact toughness value of Charpy impact specimens with dimensions of 10 mm × 10 mm × 55 mm unnotched was measured by a 150 J capacity impact testing machine (JB-300, Gaosheng, Jinan, China) at room temperature. The impact toughness values reported are the average of three samples.

**Figure 2:**
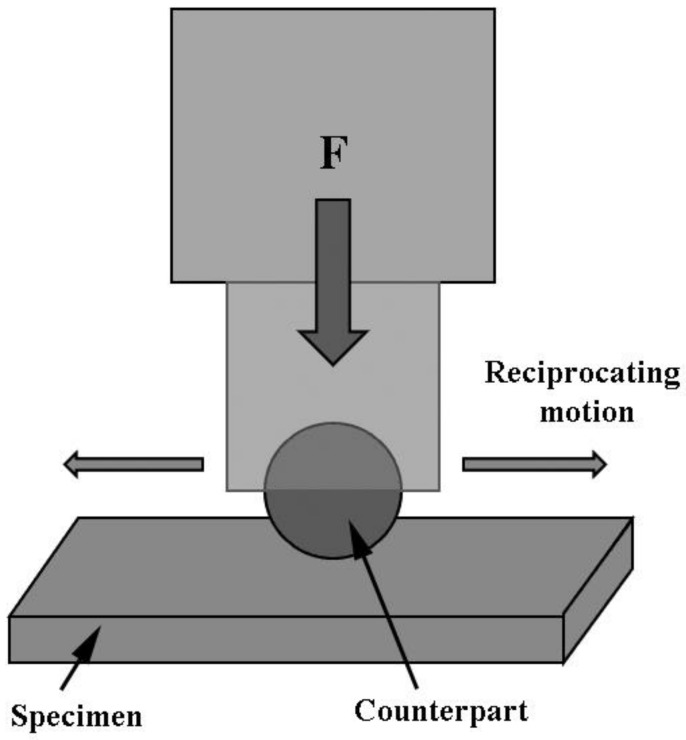
Schematic diagram of the reciprocating friction and wear tester (HSR-2M).

The wear test was carried out on a reciprocating friction and wear tester (HSR-2M, Zhongkekaihua, Lanzhou, China) with the wear condition of dry friction in an atmospheric environment. The schematic diagram of the friction and wear tester is shown in Figure 2. The counterpart selected was a SiC ball with a diameter of 6 mm. The indenter was operated at a load of 50 N at a frequency of 5 Hz. The running length was 5 mm, and the wearing time was 40 min. The cross-section areas of wear scars were measured by a three-dimensional profilometer (Nano map-500LS, AEP Technology, Santa Clara, CA, USA). Seven data were measured for each group. The wear ratio (w) was calculated by Equation (1) as follows:

(1) $$w=\frac{V}{\mathrm{SF}}$$

where V is the wear volume, which is the product of the cross-section area of the worn scar measured by the profilometer and the running length; S represents the total wear length, which is calculated by the frequency of the indenter, running length, and wearing time; and F is the load applied.

## 3. Results and Discussion

### 3.1. Microstructure

**Figure 3:**
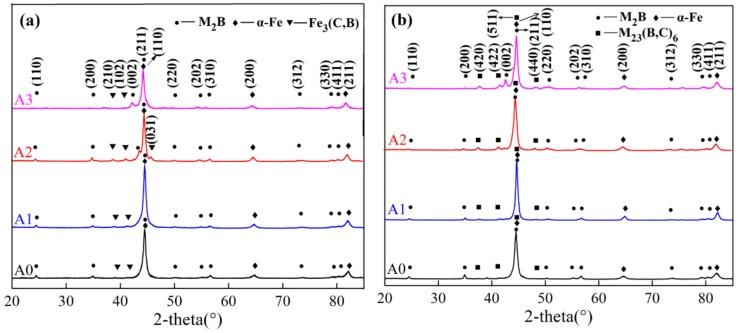
XRD results of as-cast and heat-treated samples: (**a**) as-cast alloys; (**b**) heat-treated alloys.

The XRD patterns of as-cast HBCS with different Mo contents are shown in Figure 3a. It was notable that the as-cast alloy A0 was composed of α-Fe, M_2_B (M represents Fe, Cr, and Mo), and Fe_3_(C,B). The main peak of α-Fe tended to move leftward due to the lattice distortion resulting from the larger atom radius of Mo, as displayed in Figure 3b. Besides, it is noteworthy that Fe_3_(C,B) dissolved into the matrix during heat treatment, accompanied by the precipitation of secondary phase M_23_(B,C)_6_.

**Figure 4:**
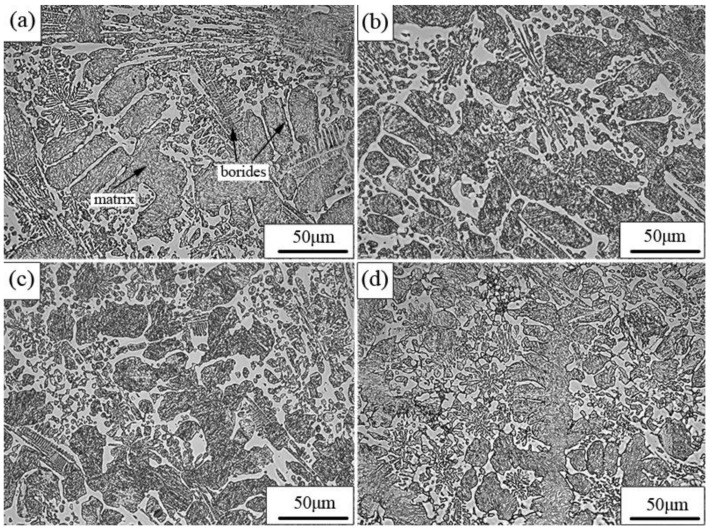
OM images of as-cast alloys: (**a**) A0; (**b**) A1; (**c**) A2; (**d**) A3.

**Figure 5:**
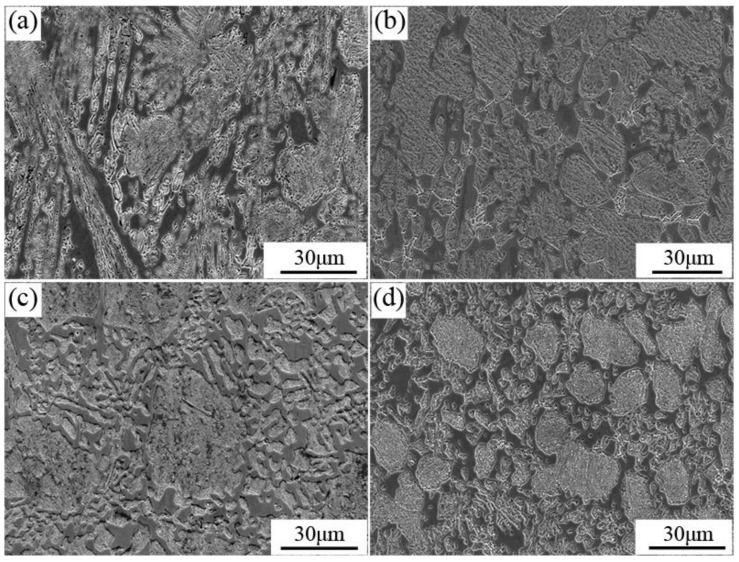
SEM images of as-cast and heat-treated alloys: (**a**) as-cast A0; (**b**) as-cast A3; (**c**) heat-treated A0; (**d**) heat-treated A3.

The as-cast microstructure consisted of dendritic matrix and inter-dendritic eutectic borides, as presented in Figure 4. With the increase of Mo content, the number of fish-bone borides decreased, while the quantity of cluster-like borides increased. Moreover, the borides tended to be refined. SEM graphs of as-cast and heat-treated samples of A0 and A3 are shown in Figure 5. It was observed that Fe_3_(B,C) and partial M_2_B dissolved in the matrix during the heat treatment, and a fine secondary phase precipitated from the matrix, which was identified as M_23_(B,C)_6_ by XRD. Compared to the as-cast condition, the edges of borides became rounded after heat treatment due to the dissolution. In addition, Mo refined the borides network, as shown in Figure 5c,d. Moreover, the size of the secondary phase M_23_(B,C)_6_ precipitated in heat treatment became smaller because of the enhancement of the thermal stability of martensite caused by Mo.

**Figure 6:**
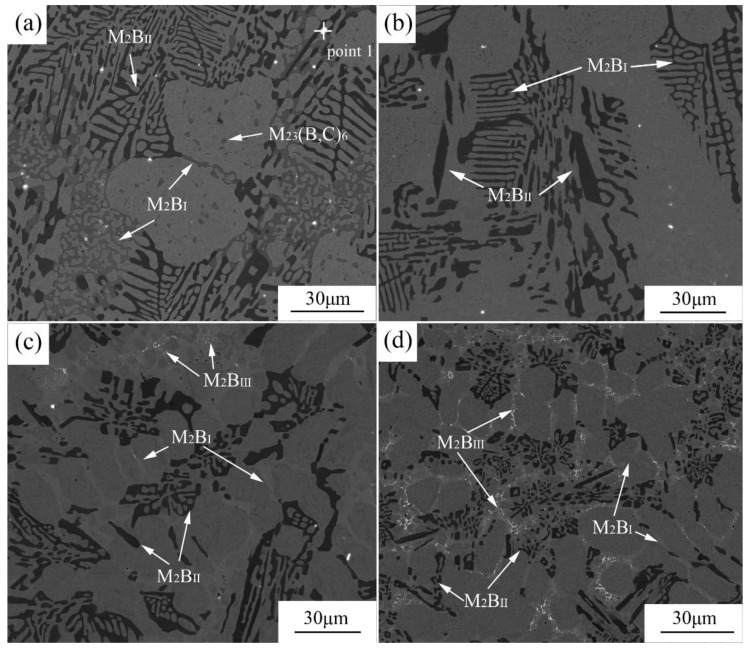
BSE images of heat-treated alloys: (**a**) A0; (**b**) A1; (**c**) A2; (**d**) A3.

**Figure 7:**
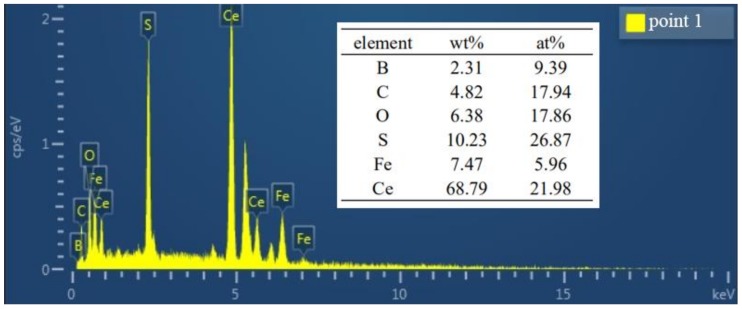
EDX results of Point 1 in Figure 6a.

The BSE experiment was carried out to further determine the element distribution of M_2_B in different samples, which are shown in Figure 6. The fish-bone M_2_B distributed in a reticular manner (denoted as M_2_B_I_ and M_2_B_II_, respectively) according to Figure 6a. The fine and dispersed phase was M_23_(B,C)_6_ precipitated within the grain during heat treatment. The bright white spots were Ce-bearing compounds confirmed by EDX in Figure 7, which could be the nuclei of grains during solidification because of the low mismatch with the γ-Fe lattice; for example, the mismatches of Ce_2_O_2_S and Ce_2_S_3_ with γ-Fe were 7% and 5%, respectively. Therefore, the primary austenite grains and borides could be refined by the addition of Ce. Moreover, Ce could promote the distribution uniformity of borides according to our previous research [35]. The formation of blocky M_2_B began when 1.04 wt.% Mo was added, and the fish-bone M_2_B still existed according to Figure 6b. When the content of Mo increased to 2.10 wt.%, the fish-bone M_2_B almost disappeared, together with the increment of M_2_B_II_ in a blocky and cluster-like shape. The morphology of M_2_B_I_ became dendritic at the junction of the network. In addition, a very fine grainy phase (marked as M_2_B_III_) was preliminarily generated at the junction of the adjacent M_2_B. When the content of Mo reached 2.86 wt.%, the number of M_2_B_III_ increased remarkably. According to Figure 6a–d, the morphology of M_2_B was improved with the increase of Mo addition, which was manifested as the almost vanishment of fish-bone M_2_B and the significantly growing quantity of M_2_B in the cluster-like and blocky morphology. The reason for the change of M_2_B morphology was inferred as Mo reducing the preferential growth tendency of Fe_2_B in some directions, which led to the significant variation of the morphology of M_2_B. Similar results were also observed in Azakli’s research [36].

**Figure 8:**
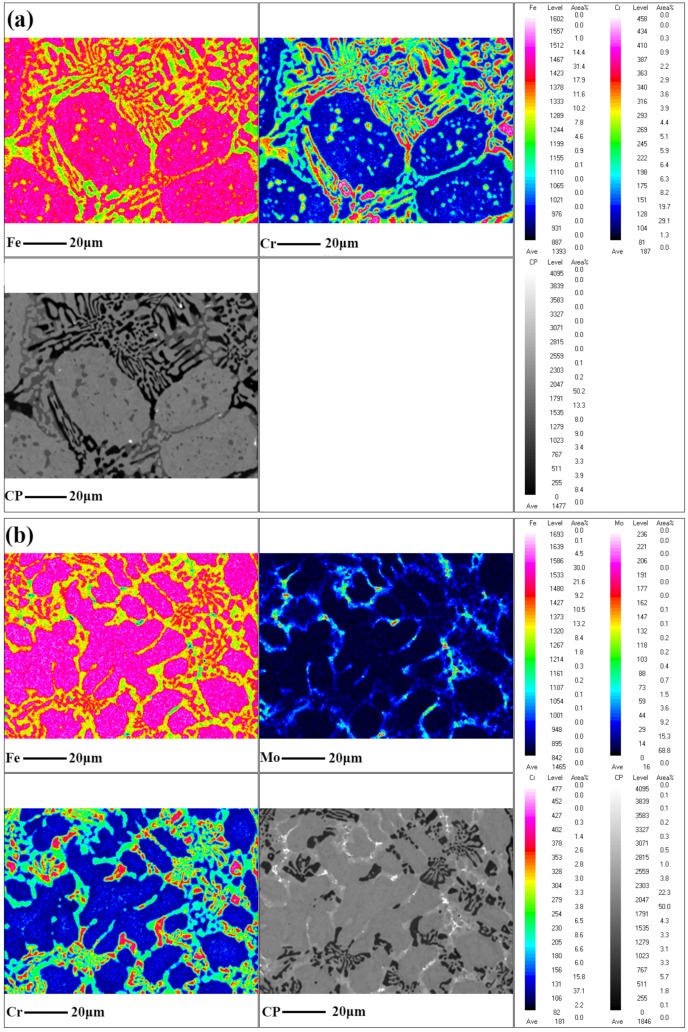
Elemental distribution mapping by EPMA: (**a**) heat-treated A0; (**b**) heat-treated A3.

To figure out the phase evolution process with Mo addition, the distribution of alloying elements and the composition of M_2_B were measured by EPMA, as shown in Figure 8. As can be seen, M_2_B was mainly composed of Fe-rich borides in grey (corresponding to M_2_B_I_) and Cr-rich borides in a darker color (corresponding to M_2_B_II_) without Mo addition, as exhibited in Figure 8a. In addition to M_2_B_I_ and M_2_B_II_, a new type of grainy boride M_2_B_III_ formed between the adjacent borides, which was in a bright white contrast at the micron scale when the content of Mo reached 2.86 wt.%, as shown in Figure 8b. Moreover, M_2_B_I_ in the reticular part became lighter with the dissolution of Mo, and the amount of M_2_B_I_ became larger. For the alloying elements Cr and Mo, it was clear that their content in M_2_B was higher than that in the matrix, as shown in Figure 8, which indicated that the two alloying elements were preferentially dissolved in borides.

The components of the borides were quantitively measured by EPMA and are displayed in Table 2. As shown in Table 2, Fe took over the greatest proportion of metallic elements in all borides. Cr, Mo, and Mn dissolved in the lattice of Fe_2_B substitutionally, and the concentration of the alloying elements was relatively low. The concentration of Fe was the highest in M_2_B_I_, the stoichiometric formula was Fe_1.91_Cr_0.10_Mo_0.07_Mn_0.03_(B,C). The stoichiometric formula of the M_2_B_II_ was measured as Fe_1.74_Cr_0.27_Mo_0.02_Mn_0.02_(B,C), while the Mo content was the lowest. The content of Mo increased significantly in M_2_B_III_ and reached 23.2 at.%, which was the highest among the three kinds of M_2_B, and the content of Fe was the lowest as a result. The stoichiometric formula of M_2_B_III_ was identified as Fe_1.24_Cr_0.10_Mo_0.71_Mn_0.02_(B,C). In addition, only a very small number of Mn dissolved in borides, and no remarkable difference was observed in the three borides, which indicated that there was no prior dissolution of Mn in M_2_B. The addition of Mo changed the solid solution degree of the alloying elements in M_2_B and led to the morphology amelioration. Besides, M_2_B was remarkably refined with the increase of Mo content. This may be the lower formation enthalpy of Mo_2_B compared with that of Fe_2_B and Cr_2_B, leading to a prior formation during solidification, which hindered the growth of eutectic borides and refined the microstructure [37]. It was also speculated that the change of bond energy in different directions of the Fe_2_B lattice was caused by the solid solution of the alloying elements, which was probably beneficial to the property of Fe_2_B [32,38].

### 3.2. Mechanical Properties

**Figure 9:**
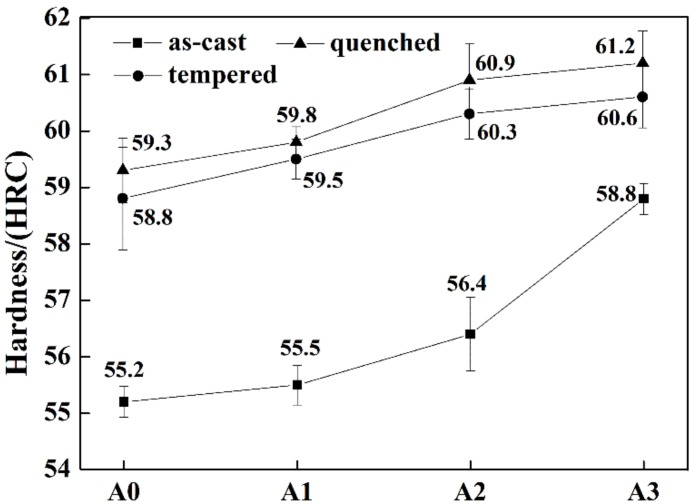
Hardness of the samples of A0, A1, A2, and A3.

**Figure 10:**
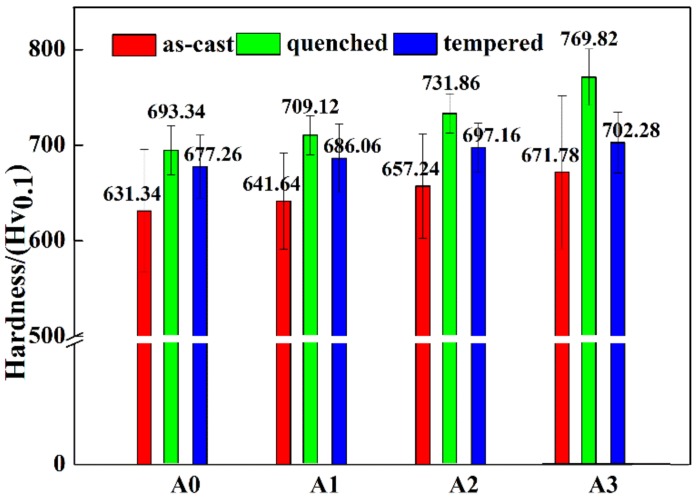
Hardness of the matrix of alloy A0, A1, A2, and A3 in as-cast, quenched, and tempered states.

The changes of hardness in as-cast, quenched, and tempered states are presented in Figure 9. The hardness of all states rose with the increase of Mo addition. In the as-cast condition, more martensite was obtained due to the improvement of hardenability caused by the solid solution of Mo, and the hardness was increased as a result. The enhancement of hardness in quenched and tempered states mainly benefitted from the solution strengthening of Mo. The effect of Mo content on matrix hardness is shown in Figure 10. The variation trend of matrix hardness was identical to that of the hardness of HBCS. According to previous research [39,40], the solution of Cr in matrix would result in lattice distortion, increasing the solid solubility of B in matrix, which improved the hardenability of the matrix. Similarly, the hardness was enhanced due to the solution of Mo.

**Figure 11:**
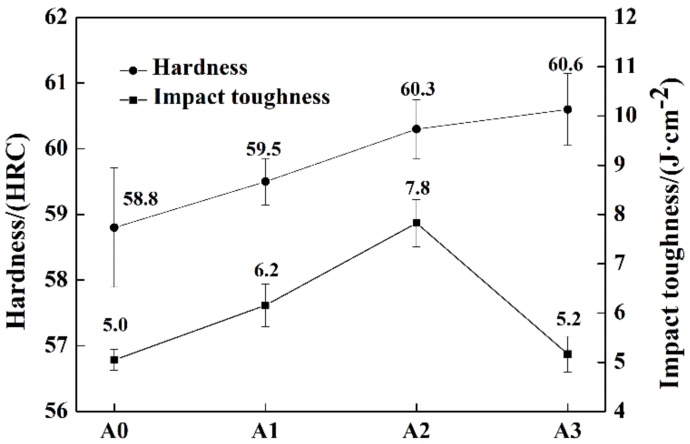
Hardness and impact toughness of heat-treated samples of alloys A0, A1, A2, and A3.

The hardness and impact toughness of tempered specimens are shown in Figure 11. The hardness of the samples was proportional to the concentration of Mo, while the impact toughness of the samples increased firstly, reaching the maximum with 2.10 wt.% Mo addition and then decreased with the further increase of Mo content. The impact toughness of the sample with the addition of 2.86 wt.% Mo was similar to that of the sample without Mo. Generally speaking, the relationship between hardness and toughness was inverse for conventional metallic materials [41]. However, the hardness and toughness of HBCS simultaneously increased in an unconventional manner when the concentration of Mo was less than 2.10 wt.%. The confusing phenomenon could be attributed to the microstructure evolution of the Mo alloyed HBCS. Firstly, the increasing Mo concentration enhanced the solid solution strengthening effect, which was beneficial to the improvement of matrix hardness. Besides, the refinement of M_2_B increased the resistance of dislocation displacement during deformation, which enhanced the strength, hardness, and toughness. The decrease of fish-bone M_2_B enhanced the impact toughness effectively according to Figure 6. Moreover, the hardness and fracture toughness of M_2_B rose with the substitution of Fe atoms by Cr and Mo atoms in the Fe_2_B lattice, referring to the previous study [28,33,37]. As a result, both the hardness and impact toughness increased with the ascending Mo concentration when it was no more than 2.10 wt.%. When the addition of Mo reached 2.86 wt.%, a large number of grainy M_2_B formed at the grain boundary, reducing the continuity of the matrix greatly, which was harmful to the impact toughness of the sample. Moreover, it was revealed by Jian [32] that the fracture toughness of M_2_B decreased when the content of Mo was 3.0 wt.% compared to that of 2.0 wt.%. Therefore, the impact toughness declined when the Mo concentration was 2.86 wt.%, even though M_2_B was further refined. Compared to the effect of Cr on the mechanical properties studied by Ma [21] and Lentz [28], the effect of Mo and Cr on the hardness of HBCS was similar, which was mainly benefited from the enhancement of the hardenability and solution strengthening effect. As for the toughness, Cr improved the fracture toughness of M_2_B, while proper addition of Mo increased the impact toughness by ameliorating the M_2_B morphology from fish-bone to cluster-like and grainy M_2_B.

**Figure 12:**
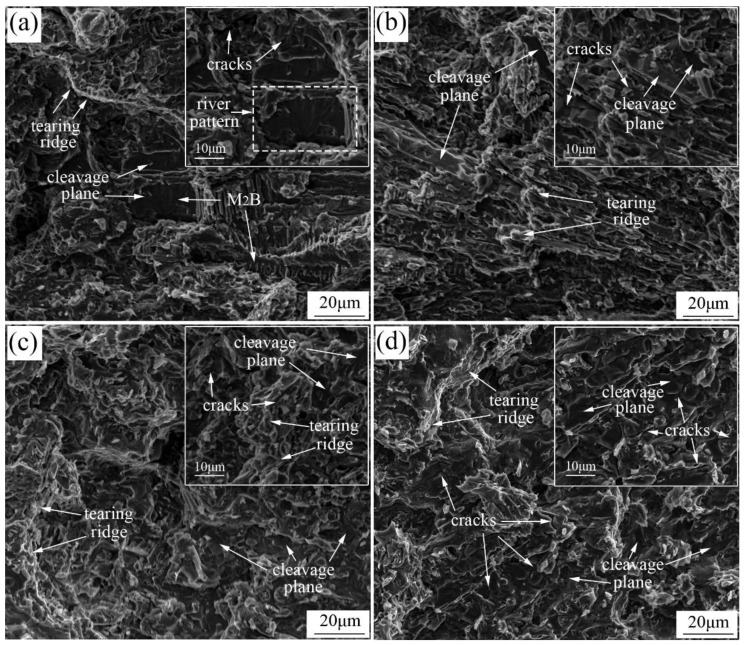
Fracture images and the corresponding magnification of heat-treated samples after the impact test: (**a**) A0; (**b**) A1; (**c**) A2; (**d**) A3.

The impact fracture morphology is shown in Figure 12. Extensive cleavage planes are observed in Figure 12a–d, which are identified by the typical river patterns shown in the magnified figures at the upper right corner of Figure 12a. The cleavage fracture was attributed to the intrinsic brittleness of Fe_2_B caused by the weak B-B bond along the [002] direction [26], which led to the easier process of the initiation and propagation of cracks. The matrix fractured in a plastic form, as presented in Figure 12a–d. Plenty of tearing ridges were observed around the cleavage planes, indicating the better toughness of the matrix compared to M_2_B. It could be concluded that the fracture of all four groups of samples was quasi-cleavage. In addition, the cleavage planes in a large size observed in Figure 12a,b indicated that the cleavage fracture of the massive fish-bone M_2_B was harmful to the impact toughness of HBCS. Because of the refinement of M_2_B and the elimination of fish-bone M_2_B, the large-sized cleavage planes were replaced by a series of small cleavage planes, as shown in Figure 12c,d. However, when the content of Mo was 2.86 wt.%, the extensive formation of grainy M_2_B at grain boundary reduced the continuity of the matrix, which led to the decrease of plastic deformation and the reduction of impact toughness. Moreover, the decline of the fracture toughness of M_2_B when the Mo concentration reached 2.86 wt.% as mentioned above, accelerated the rate of crack initiation and propagation in M_2_B. More cracks are observed in the magnification in Figure 12d. The impact toughness descended as a result when the Mo content was 2.86 wt.%. Those were well consistent with the experimental results of impact toughness in Figure 11.

### 3.3. Tribological Properties

**Figure 13:**
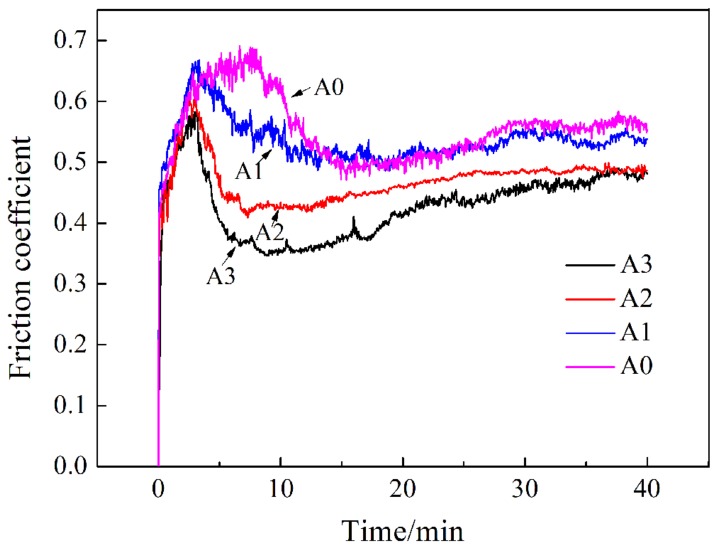
Friction coefficient of the heat-treated samples in the wear test.

The friction coefficient of the samples is shown in Figure 13. As exhibited in Figure 13, with time prolonging, the friction coefficients of all the samples presented a similar trend that started with a dramatic increase, then followed by some reduction, and finally showed a slow and continuous rise. The reason for this change was that the samples would undergo a grinding-in period, which depended on the surface roughness, temperature, humidity, and so on. After a certain period of pre-grinding, the friction force and the roughness of the grinding surface tended to be steady, and then, it would go through the steady-wear stage. The friction required in the grinding-in period was greater than that in the steady-wear stage, which led to a relatively higher friction coefficient. With the ongoing of the steady-wear stage, the friction coefficient was slowly increased, which was attributed to the damage to the worn surface. Comparing the friction coefficient-time curves of all the samples, it could be concluded that the average friction coefficient of the steady-wear stage decreased with the increase of Mo content. The depth, width, and cross-sectional area of the wear scars were measured by the profilometer, as shown in Table 3. The parameters presented a tendency of declining with the increasing addition of Mo, indicating a slighter abrasion process and better wear resistance. As a result, the friction coefficient of the steady-wear stage was relatively lower when the Mo concentration was higher.

**Figure 14:**
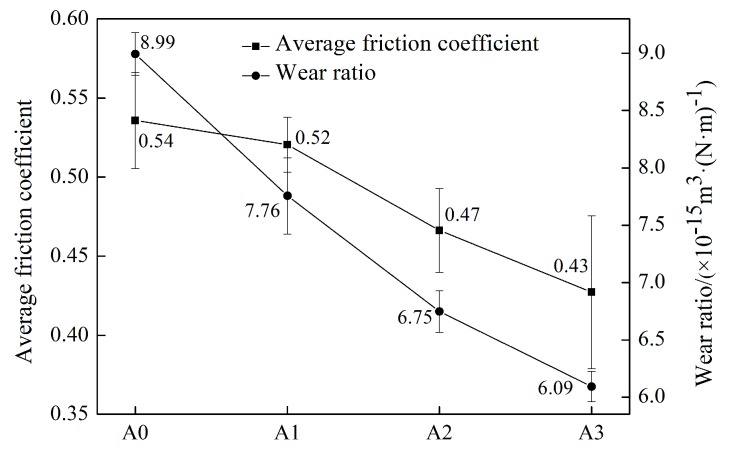
Average friction coefficient and wear ratio of the heat-treated samples.

The wear ratio calculated by Equation (1) and the average friction coefficient in the steady-wear stage are shown in Figure 14. The average friction coefficient decreased with the increment of Mo content, and the wear ratio showed a descending tendency. It is well known that the excellent wear resistance depends on the good coordination between the matrix and hard phase [42]. According to the mechanical properties mentioned before, the hardness of matrix increased with the addition of Mo. On account of a synchronous wear process of the matrix and borides, the matrix with higher hardness possessed a better resistance to deformation and provided stronger support to the hard phase. Thus, the A3 sample showed better wear resistance. On the contrary, when the hardness of the matrix was lower, easier deformation occurred during wear process. Without the support of the matrix, borides would crumble and then be peeled off easily by the abrasive, which in turn would lead to further abrasion of the matrix. Such vicious cycles accelerated the abrasion process rapidly and brought about the poor wear resistance. The more severe wear process led to the higher friction coefficient when the content of Mo was lower.

**Figure 15:**
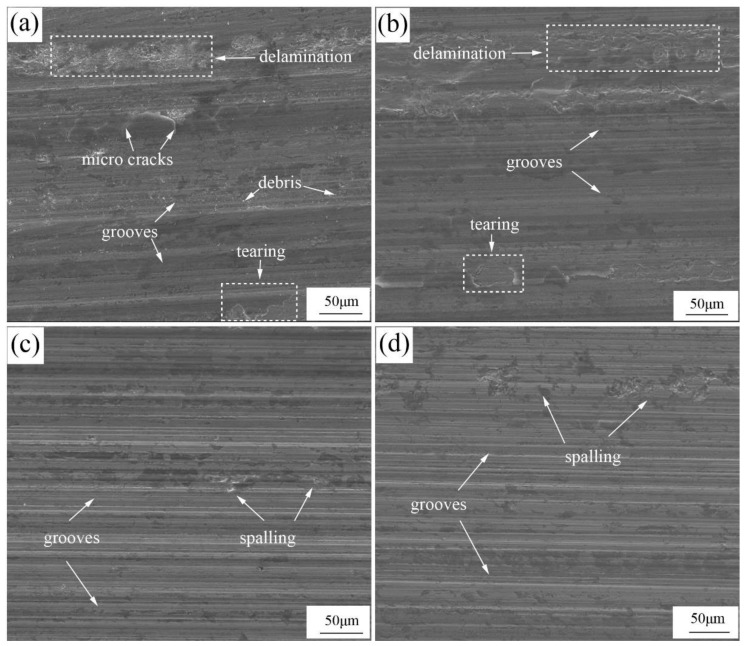
SEM images of the worn surfaces: (**a**) alloy A0; (**b**) alloy A1; (**c**) alloy A2; (**d**) alloy A3.

The wear morphology was measured by SEM, as shown in Figure 15. It can be seen that the worn surface of alloys A0 and A1 shown in Figure 15a,b had a similar wear morphology: parallel grooves, tearing, delamination, and spalling, as marked out, indicating the characteristics of abrasive wear and adhesive wear. For alloys A2 and A3, the morphology of the worn surface changed remarkably with the increasing Mo addition, as shown in Figure 15c,d: the delamination and tearing phenomena disappeared, and only a series of parallel grooves and a few pits led by spalling were observed, demonstrating the characteristic of abrasive wear. Due to the relatively soft matrix of alloys A0 and A1, penetration of the abrasive was deeper, which led to the removal of the debris under the force parallel to the worn surface. The support to borides reduced significantly owing to the severe abrasion of the matrix, resulting in the breakdown of borides, which triggered the further abrasion rapidly. A more severe wear process is observed in Figure 15a,b. Thus, the wear mechanisms of alloys A0 and A1 were abrasive wear and adhesive wear. However, because of the higher resistance to the abrasive, the matrix of alloys A2 and A3 provided a stronger support to M_2_B, which enhanced the wear resistance effectively. Only a few M_2_B collapsed and broke down under the vertical force due to the low toughness and were peeled off from the surface, leaving pits of different sizes. The wear mechanism of alloys A2 and A3 was abrasive wear. The increase of the matrix hardness caused by Mo addition greatly promoted the improvement of wear resistance.

## 4. Conclusions

The microstructure evolution and properties of HBCS with different Mo contents were systematically studied. The main results are listed as follows:

(1) Mo promoted the formation of Mo-rich and Cr-rich M_2_B. Moreover, with the increase of Mo concentration, the morphology of borides changed from the fish-bone Fe-rich M_2_B to cluster-like and blocky Cr-rich M_2_B and grainy Mo-rich M_2_B. Meanwhile, borides were greatly refined.
(2) The hardness of HBCS increased with the increase of Mo content, which was mainly affected by the solid solution strengthening of Mo and the refinement of M_2_B.
(3) Although all four alloys showed quasi-cleavage, the impact toughness rose firstly, then reduced with the increase of Mo content, and reached its maximum value at 2.10 wt.% Mo. The decrease of fish-bone M_2_B and the refinement of M_2_B were responsible for the improvement of impact toughness. However, the extensive formation of Mo-rich M_2_B at the grain boundary and the decrease of fracture toughness of M_2_B greatly damaged the impact toughness when the Mo content was 2.86 wt.%.
(4) The average friction coefficient and wear ratio both showed a descending trend with the increasing Mo content. The wear mechanism changed from abrasive wear and adhesive wear to abrasive wear when the content of Mo exceeded 2.10 wt.% due to the better resistance of the matrix to the abrasive.

## Figures and Tables

**Table 1:** Chemical composition of the alloys (wt.%).

Alloy	B	C	Mo	Cr	Mn	Si	Ce	Fe
A0	2.29	0.42	0.00	3.87	0.76	0.48	0.09	Bal.
A1	2.24	0.48	1.04	4.07	0.85	0.51	0.13	Bal.
A2	2.22	0.43	2.10	4.10	0.82	0.53	0.08	Bal.
A3	2.16	0.47	2.86	3.79	0.75	0.47	0.11	Bal.

**Table 2:** Chemical formula of borides in different morphologies of Sample A3 by electron probe micro-analyzer (EPMA).

Eutectic M_2_B	Element (at.%)						Calculated Formula	
	B	C	Mo	Cr	Mn	Fe		
**M_2_B_I_**	**24.03**	8.16	2.27	3.18	0.82	61.54	Fe_1.91_Cr_0.10_Mo_0.07_Mn_0.03_(B,C)	M_2.11_(B,C)
M_2_B_II_	29.03	3.64	0.69	8.98	0.75	56.91	Fe_1.74_Cr_0.27_Mo_0.02_Mn_0.02_(B,C)	M_2.06_(B,C)
M_2_B_III_	28.34	4.22	23.20	3.25	0.69	40.30	Fe_1.24_Cr_0.10_Mo_0.71_Mn_0.02_(B,C)	M_2.07_(B,C)

**Table 3:** Parameters of the worn scars.

Alloy	Depth (μm)	Width (mm)	Area (μm^2^)
A0	28.94	1.03	10790
A1	21.32	0.84	9308
A2	17.44	0.77	8097
A3	14.82	0.75	7313

## References

[1] Luo Q., Xie J., Song Y. (1995). Effects of microstructures on the abrasive wear behaviour of spheroidal cast iron. Wear.

[2] Cetinkaya C. (2006). An investigation of the wear behaviours of white cast irons under different compositions. Mater. Des..

[3] Lv H., Zhou R., Li L., Ni H., Zhu J., Feng T. (2018). Effect of Electric Current Pulse on Microstructure and Corrosion Resistance of Hypereutectic High Chromium Cast Iron. Materials.

[4] Shimizu K., Kusumoto K., Yaer X., Zhang Y., Shirai M. (2017). Effect of Mo content on erosive wear characteristics of high chromium cast iron at 1173 K. Wear.

[5] Medyński D., Samociuk B., Janus A., Chęcmanowski J. (2019). Effect of Cr, Mo and Al on Microstructure, Abrasive Wear and Corrosion Resistance of Ni-Mn-Cu Cast Iron. Materials.

[6] Park J.S. (2008). The key role of forging in ancient steel making from white cast iron. Mater. Charact..

[7] Kowalska J., Ryś J., Cios G., Bednarczyk W. (2019). The effect of reduced temperatures on microstructure development in tensile tested high-manganese steel. Mater. Sci. Eng. A.

[8] Jinzhu L., Yongfa M. (1993). Development of abrasion-resistant Ni-hard 4 cast irons. Wear.

[9] Liujie X., Jiandong X., Shizhong W., Yongzhen Z., Rui L. (2006). Investigation on wear behaviors of high-vanadium high-speed steel compared with high-chromium cast iron under rolling contact condition. Mater. Sci. Eng. A.

[10] Fernández I., Belzunce F.J. (2008). Wear and oxidation behaviour of high-chromium white cast irons. Mater. Charact..

[11] Liu B., Qin T., Xu W., Jia C., Wu Q., Chen M., Liu Z. (2019). Effect of Tempering Conditions on Secondary Hardening of Carbides and Retained Austenite in Spray-Formed M42 High-Speed Steel. Materials.

[12] Huang Z., Xing J., Guo C. (2010). Improving fracture toughness and hardness of Fe_2_B in high boron white cast iron by chromium addition. Mater. Des..

[13] Chen X., Li Y., Zhang H. (2011). Microstructure and mechanical properties of high boron white cast iron with about 4 wt% chromium. J. Mater. Sci..

[14] Moon H.K., Lee K.B., Kwon H. (2008). Influences of Co addition and austenitizing temperature on secondary hardening and impact fracture behavior in P/M high speed steels of W–Mo–Cr–V (–Co) system. Mater. Sci. Eng. A.

[15] Li Y., Li P., Wang K., Li H., Gong M., Tong W. (2018). Microstructure and mechanical properties of a Mo alloyed high chromium cast iron after different heat treatments. Vacuum.

[16] Li F., Li Z. (2014). Study on improvement of hard phase morphology and properties of hypoeutectic Fe–C–B alloy. J. Alloys. Compd..

[17] Spiridonova I.M. (1984). Structure and properties of iron-boron-carbon alloys. Met. Sci. Heat. Treat..

[18] Egorov M.D., Sapozhnikov Y.L., Shakhnazarov Y.V. (1989). Effect of carbon content on the structure, hardness, and thermal stability of boron-chromium cast steels. Met. Sci. Heat. Treat..

[19] Christodoulou P., Calos N. (2001). A step towards designing Fe–Cr–B–C cast alloys. Mater. Sci. Eng. A.

[20] Liu Z., Li Y., Chen X., Hu K. (2008). Microstructure and mechanical properties of high boron white cast iron. Mater. Sci. Eng. A.

[21] Ma S., Xing J., Liu G., Yi D., Fu H., Zhang J., Li Y. (2010). Effect of chromium concentration on microstructure and properties of Fe–3.5 B alloy. Mater. Sci. Eng. A.

[22] Chen X., Li Y. (2010). Effect of heat treatment on microstructure and mechanical properties of high boron white cast iron. Mater. Sci. Eng. A.

[23] Jahazi M., Jonas J.J. (2002). The non-equilibrium segregation of boron on original and moving austenite grain boundaries. Mater. Sci. Eng. A.

[24] Fu H., Xiao Q., Kuang J., Jiang Z., Xing J.D. (2007). Effect of rare earth and titanium additions on the microstructures and properties of low carbon Fe–B cast steel. Mater. Sci. Eng. A.

[25] Jian Y., Huang Z., Xing J., Gao Y. (2018). Effects of chromium on the morphology and mechanical properties of Fe_2_B intermetallic in Fe-3.0 B alloy. J. Mater. Sci..

[26] Li M., Fu S., Xu W. (1995). Valence electron structure of Fe_2_B phase and its eigen-brittleness. Acta. Metall. Sin..

[27] Wang Y., Sun J., Jiang T., Sun Y., Guo S., Liu Y. (2018). A low-alloy high-carbon martensite steel with 2.6 GPa tensile strength and good ductility. Acta. Mater..

[28] Lentz J., Röttger A., Großwendt F., Theisen W. (2018). Enhancement of hardness, modulus and fracture toughness of the tetragonal (Fe, Cr)_2_B and orthorhombic (Cr, Fe)_2_B phases with addition of Cr. Mater. Des..

[29] Wei X., Chen Z., Zhong J., Wang L., Yang W., Wang Y. (2018). Effect of alloying elements on mechanical, electronic and magnetic properties of Fe2B by first-principles investigations. Comput. Mater. Sci..

[30] Yi Y., Xing J., Lu Y., Gao Y., Fu H., Yu L., Wan M., Zheng Q. (2018). Effect of normal load on two-body abrasive wear of an Fe-B-Cr-C based alloy with minor Cu and Ni additions. Wear.

[31] Yi Y., Xing J., Wan M., Yu L., Lu Y., Jian Y. (2017). Effect of Cu on microstructure, crystallography and mechanical properties in Fe-B-C-Cu alloys. Mater. Sci. Eng. A.

[32] Jian Y., Huang Z., Xing J., Guo X., Jiang K. (2017). Effect of molybdenum addition on mechanical properties of oriented bulk Fe_2_B crystal. J. Mater. Res..

[33] Huang Z., Xing J., Tao X. (2012). Effect of molybdenum addition on fracture toughness and hardness of Fe_2_B in Fe–B–C cast alloy. Int. J. Mater. Res..

[34] Yi Y., Xing J., Ren X., Fu H., Li Q., Yi D. (2019). Investigation on abrasive wear behavior of Fe-B alloys containing various molybdenum contents. Tribol. Int..

[35] Yong X., Zhiguo C., Xiang W., Zhongjia W. (2015). Influence of Ce on microstructure and properties of high-carbon high-boron steel. Rare. Met. Mater. Eng..

[36] Azakli Y., Cengiz S., Tarakci M., Gencer Y. (2016). Characterisation of boride layer formed on Fe–Mo binary alloys. Surf. Eng..

[37] Wei X., Chen Z., Zhong J., Wang L., Wang Y., Shu Z. (2018). First-principles investigation of Cr-doped Fe_2_B: Structural, mechanical, electronic and magnetic properties. J. Magn. Magn. Mater..

[38] Jian Y., Huang Z., Xing J., Wang B. (2015). Effects of chromium addition on fracture toughness and hardness of oriented bulk Fe_2_B crystals. Mater. Charact..

[39] Liu Z., Chen X., Li Y., Hu K. (2008). Effect of chromium on microstructure and properties of high boron white cast iron. Metall. Mater. Trans. A.

[40] Guo C., Kelly P.M. (2003). Boron solubility in Fe–Cr–B cast irons. Mater. Sci. Eng. A.

[41] Liu T., Cao Z., Wang H., Wu G., Jin J., Cao W. (2020). A new 2.4 GPa extra-high strength steel with good ductility and high toughness designed by synergistic strengthening of nano-particles and high-density dislocations. Scr. Mater..

[42] Shah M., Bakshi S.D. (2018). Three-body abrasive wear of carbide-free bainite, martensite and bainite-martensite structure of similar hardness. Wear.

